# The Association of CYP17A1, CYP19A1, and SHBG Gene Polymorphisms in Polycystic Ovary Syndrome Susceptibility: A Systematic Review and Meta-Analysis

**DOI:** 10.3389/fphys.2022.741285

**Published:** 2022-05-09

**Authors:** Chuan Xing, Han Zhao, Jiaqi Zhang, Bing He

**Affiliations:** The First Endocrine Department of Shengjing Hospital of China Medical University, Shenyang, China

**Keywords:** polycystic ovary syndrome, single nucleotide polymorphism, CYP19, CYP17, sex hormone binding globulin

## Abstract

**Objective:** To elucidate the relationship between CYP17A1/CYP19A1/SHBG gene polymorphisms and PCOS susceptibility.

**Methods:** We searched multiple databases from inception to December 2020 and meta analysis was conducted to elucidate the relationship between gene polymorphisms and PCOS risk.

**Results:** 26 studies were included, comprising 4860 PCOS and 4043 controls. CYP17A1 rs743572 polymorphisms were found to be negatively associated with PCOS risk under dominant model (*p* = 0.017, OR = 0.83, 95%CI 0.72–0.97, *I* ^*2*^ = 74.80%, *P*_*heterogeneity*_ = 0.000) in the general population while neither CYP19A1 rs2414096 polymorphisms (*p* = 0.578, OR = 0.87, 95%CI 0.54–1.41, *I* ^*2*^ = 95.90%, *P*_*heterogeneity*_ = 0.000) nor SHBG rs6529 polymorphisms (*p* = 0.752, OR = 0.99, 95%CI 0.94–1.05, *I* ^*2*^ = 60.90%, *P*_*heterogeneity*_ = 0.012) was associated with PCOS susceptibility under dominant model in the general population.

**Conclusion:** CYP17A1 rs7435721 polymorphisms might be protective factors against PCOS in general populations.

**Systematic Review Registration**: https://www.crd.york.ac.uk/prospero/#myprospero, identifier CRD4202122640.

## Introduction

Polycystic ovary syndrome (PCOS) is a common reproductive endocrine disorder that occurs in approximately 5–10% of the women of childbearing age (Goodarzi et al., 2011; Dadachanji et al., 2018). PCOS is typically characterized by chronic anovulation, elevated androgen levels, a distorted luteinizing hormone/follicle-stimulating hormone (LH/FSH) ratio, irregular menstrual cycle, appearance of polycystic ovaries, and insulin resistance (Goodarzi et al., 2011).

Hyperandrogenemia is a key indicator of PCOS, and increased androgen concentrations including testosterone (T) and androstenedione have been observed in most patients, while increased dehydroepiandrosterone sulfate has been observed in a minority [∼25%] of the patients (Livadas et al., 2014). Androgens are produced in the ovaries and adrenal glands as the final products of a series of enzymatic reactions involving the conversion of cholesterol into dehydroepiandrosterone and androstenedione. In both locations, the rate of sex steroid synthesis is limited by certain crucial enzymes (Wawrzkiewicz-Jałowiecka et al., 2020). The heterogeneity of the androgen phenotype in the steroid synthesis pathway may be attributed to differences in the activity of important enzymes; for example, in hyperandrogenic PCOS patients, the activities of 17 and 20 lyases and 3β-hydroxysteroid dehydrogenase II (3β-HSD) increase, while aromatase activity bound to the Δ4 pathway decreases (de Medeiros et al., 2015). Mutations in steroid pathway genes, such as CYP1A, CYP19, CYP17, CYP3, CYP11, and CYP21, may affect androgen synthesis (de Medeiros et al., 2015; Ajmal et al., 2019). As a transporter of sex hormones, sex hormone binding globulin (SHBG), produced in the liver, combines with circulating steroids with a high affinity to regulate the bioavailability and concentration of bioactive sex hormones in the blood (Hammond et al., 2012). As SHBG shows a high affinity for T and low affinity for estradiol, it can effectively regulate the levels of bioactive free T in the body (Somboonporn and Davis, 2004). T has no biological effect when combined with SHBG, and only approximately 1–2% of the total T has biological activity in normal women. Therefore, SHBG can be used to judge the severity of hyperandrogenemia and evaluate the therapeutic effect in PCOS women (Zhu et al., 2019).

Previous genetic association studies have found that a large number of genetic variations are related to PCOS susceptibility, and genetic factors may greatly impact the occurrence of PCOS (Saddick, 2020). Single nucleotide polymorphisms (SNPs) might reveal functional changes caused by amino acid variation or gene expression regulation. Candidate gene investigations provide insight into different frequency distributions in healthy and diseased populations (Douma et al., 2019); however, previous studies exploring the potential relationship between PCOS susceptibility and CYP17A1/CYP19A1/SHBG gene polymorphisms used statistically insufficient samples sizes and reported inconsistent findings (Li et al., 2012; Liao and Cao, 2020; Li et al., 2021; Sharma et al., 2021). Thus, The search acronym (PICO) for our meta-analysis to elucidate the relationship between CYP17A1/CYP19A1/SHBG gene polymorphisms in both wild type and mutant type and PCOS susceptibility in general populations in a larger study cohort.

## Materials and Methods

### Literature Search

This meta-analysis was registered with the PROSPERO international prospective register of systematic reviews (registration number CRD42021226402). We searched medical literature for relevant studies using PubMed, EMBASE, the Cochrane Library, Web of Science, WanFang Database, and China National Knowledge Infrastructure (CNKI) from their date of establishment to December 2020. A total of 463 records were identified using electronic search strategies; in addition, one relevant record was obtained from the reference lists of the included studies. We used the search terms: “Steroid 17-alpha-Hydroxylase or CYP17,” “aromatase or CYP19,” “sex hormone binding globulin or SHBG,” “Single nucleotide polymorphism (SNP) or polymorphisms or genotype or genetic or mutation or variant,” and “Polycystic ovary syndrome or PCOS.” We limited the publication type to case-control studies, and there were no language or location restrictions. We also tried to search in grey literature, but no new relevant cohort studies were found.

### Inclusion and Exclusion Criteria

To be included in this meta-analysis, the studies needed to meet the following inclusion criteria: (a) originated from a case-control study design; (b) reported the cases of PCOS patients diagnosed with one of the following three diagnostic criteria: National Institutes of Health (NIH) 1999, Rotterdam 2003, and AE-PCOS Society 2006; (c) evaluated the association between SHBG or CYP17 or CYP19 gene polymorphisms and PCOS; (d) reported odds ratios (ORs) and corresponding 95% confidence intervals (CIs) or provided the distribution of sufficient genotypic and allelic data for estimation in cases and controls.

Exclusion criteria included the following: (a) insufficient data on genotyping; (b) no control population; (c) study included disorders other than PCOS; (d) case reports, case series, abstracts, reviews, meta-analysis, comments, editorial articles, or letters without original data; (e) duplicate data; (f) the SNP was reported in less than 5 case-control study.

### Data Extraction and Quality Assessment

For published articles, two investigators (CX and HZ) independently extracted data and assessed methodological quality. Data were extracted from the included studies to collect the following necessary information: first author, year of publication, country of origin, ethnicity, number of cases and controls, PCOS diagnostic criteria, genotype method, genotype data, and evidence of Hardy-Weinberg equilibrium (HWE). Genotype data of case and control studies were extracted to calculate the OR with 95% CI and *p*-value of HWE in the control group. As described elsewhere, quality, internal validity, and risk of bias of the included studies were assessed using the validated quality of genetic association studies checklist (Q-Genie) (Santana et al., 2020). The Q-genie tool consists of 11 questions, which address the following aspects of study methodologies: study rationale, outcome, comparability, exposure, bias, sample size, analyses, statistical methods and control for confounding, inferences for genetic analyses, and inferences drawn from results. Each question has seven possible answers as follows: “1 (poor),” “2,” “3 (good),” “4,” “5 (very good),” “6,” “7 (excellent).” The overall quality of studies is classified as “poor quality” if score is <35, a score of 36–45 indicates “moderate quality” and a score of >45 indicates “good quality.” Discrepancies were discussed or adjudicated with the third author (H.B.), until a satisfactory consensus was reached.

### Statistical Analysis

Pooled ORs with corresponding 95% CIs were calculated to identify the potential association between susceptibility to PCOS and gene polymorphisms for the following genotypic models: dominant model (mtmt + wtmt vs. wtwt), recessive model (mtmt vs. wtwt + wtmt), co-dominant model (mtmt vs. wtwt) or (wtmt vs. wtwt), and complete overdominant model (mtmt + wtwt vs. wtmt) for rs743572 of CYP17 gene polymorphisms, rs2414096 of CYP19 gene polymorphisms, rs6529 of SHBG gene polymorphisms. In order to synthesize the best genetic model, we refer to the method of screening the best genetic model in the study of Thakkinstian et al. (2005). Data from each study is extracted as the number of subjects with each genotype (AA, Aa, and aa) in the case and control groups. The gene effects of each study are defined as OR, which are OR1, OR2, and OR3 for AA versus aa, Aa versus aa, and AA versus Aa, respectively. The pooled ORs are calculated by the inverse variance method. To determine the overall gene effect, the model that includes gene is compared with the model without gene. If the overall gene effect is statistically significant, further comparisons of OR1, OR2, and OR3 are explored. These comparisons should be performed using residual variance obtained from the regression model. These pairwise differences can be used to indicate the most appropriate genetic model, as outlined below (assuming that the risk allele is A): (a) If OR1 = OR3 = 1 and OR2 = 1, then a recessive model is suggested; (b) If OR1 = OR2 = 1 and OR3 = 1, then a dominant model is suggested; (c) If OR2 = 1 = OR3 = 1 and OR1 = 1, then a complete overdominant model is suggested; (d) If OR1 > OR2 > 1 and OR1 > OR3 > 1 (or OR1 < OR2 < 1 and OR1 < OR3 < 1), then a co-dominant model is suggested. The heterogeneity between the included studies was estimated using the Q test and the I-squared statistic. Values of *p* < 0.1 and *I* ^*2*^ > 50% were considered to indicate significant heterogeneity (Higgins et al., 2003), using of the quality effects models. Subgroup analysis was performed for gene with more than five different studies according to PCOS, control, and ethnicity. LFK index and Doi plots were used to statistically assess publication bias (Furuya-Kanamori et al., 2018). For all tests, *p* < 0.05 was considered statistically significant. Stata version 16.0 (Stata Corp, College Station, TX) was used to perform the statistical analysis.

## Results

### Literature Identification

**FIGURE 1:**
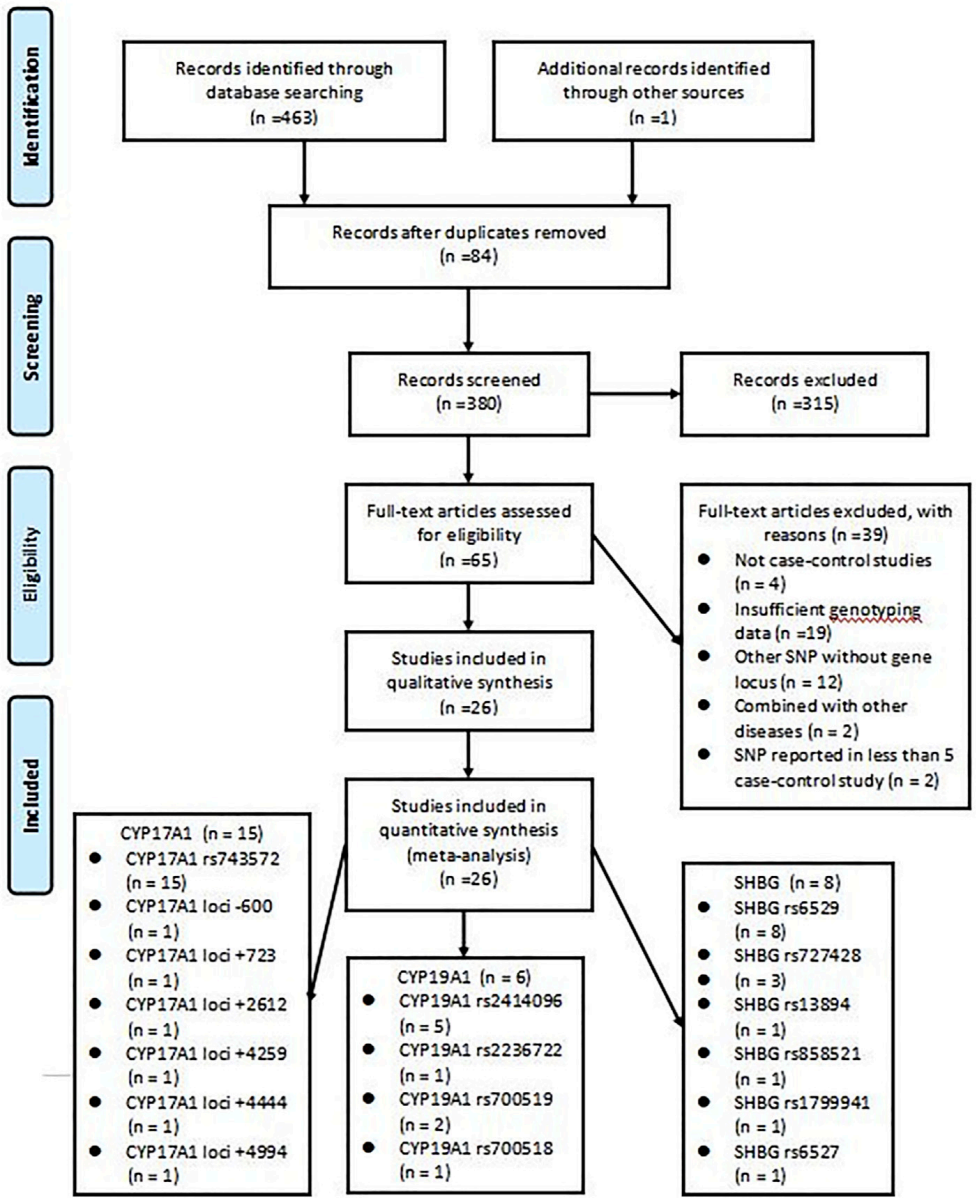
Flow diagram of studies identified in the systematic review.

The initial search strategy retrieved a total of 464 studies from all electronic databases. After reading the titles, abstracts, and full texts, we selected 26 studies (Diamanti-Kandarakis et al., 1999; Marszalek et al., 2001; Cousin et al., 2004; Kahsarmiller et al., 2004; Tan et al., 2005; Bendlová et al., 2007; Ferk et al., 2007; Echiburú et al., 2008; Park et al., 2008; Jin et al., 2009; Unsal et al., 2009; Martinez-Garcia et al., 2012; Dasgupta et al., 2014; Dai et al., 2015; Li et al., 2015; Abu-Hijleh et al., 2016; Banerjee et al., 2016; Mehdizadeh et al., 2017; Wu et al., 2017; Jiao et al., 2018; Kaur et al., 2018; Bhatnager et al., 2019; Liu et al., 2019; Rahimi and Mohammadi, 2019; Munawar Lone et al., 2020; Ashraf et al., 2021), which included a total of 8,903 women, for inclusion in the analysis. The remaining studies were excluded: 84 were duplicates, 315 were of apparent irrelevance based on a review of abstracts and titles, four were not case-control studies, 19 lacked sufficient genotyping data, 12 included other SNPs without gene locus, two were combined with other diseases, and two had SNPs reported in less than 5 case-control study. The literature screening process and results are shown in Figure 1, followed the PRISMA flowchart.

### Characteristics of Included Studies

Table 1 presents the characteristics of included studies. During the search no language restriction was applied. Trials were performed in European, American, and Asian countries and were published from 1999 to 2020. Participants in these trials were recruited from outpatient clinics, hospitals, or medical centers and were diagnosed with PCOS according to either the NIH or Rotterdam criteria. Participants aged 18–49 years were included in all 26 studies exploring the association between CYP17A1 (Diamanti-Kandarakis et al., 1999; Marszalek et al., 2001; Kahsarmiller et al., 2004; Tan et al., 2005; Echiburú et al., 2008; Park et al., 2008; Unsal et al., 2009; Dasgupta et al., 2014; Li et al., 2015; Banerjee et al., 2016; Wu et al., 2017; Kaur et al., 2018; Rahimi and Mohammadi, 2019; Munawar Lone et al., 2020; Ashraf et al., 2021), CYP19A1 (Jin et al., 2009; Mehdizadeh et al., 2017; Jiao et al., 2018; Kaur et al., 2018; Munawar Lone et al., 2020), and SHBG (Cousin et al., 2004; Bendlová et al., 2007; Ferk et al., 2007; Martinez-Garcia et al., 2012; Dai et al., 2015; Abu-Hijleh et al., 2016; Bhatnager et al., 2019; Liu et al., 2019) gene polymorphisms and PCOS risk. There were 15 studies of CYP17A1 gene polymorphisms, including 15 studies of CYP17A1 rs743572 and one study of CYP17A1 loci -600, CYP17A1 loci +723, CYP17A1 loci +2612, CYP17A1 loci +4259, CYP17A1 loci +4444 and CYP17A1 loci +4994; there were six studies of CYP19A1 gene polymorphisms, including five studies of CYP19A1 rs2414096, one study of CYP19A1 rs2236722, two studies of CYP19A1 rs700519 and one study of CYP19A1 rs700518; there were eight studies of SHBG gene polymorphisms, including eight studies of SHBG rs6529, three studies of SHBG rs727428, one study of SHBG rs13894, one study of SHBG rs858521, one study of SHBG rs1799941 and one study of SHBG rs6527. There were 17 studies of Asian patients (Tan et al., 2005; Park et al., 2008; Jin et al., 2009; Unsal et al., 2009; Dasgupta et al., 2014; Dai et al., 2015; Li et al., 2015; Banerjee et al., 2016; Mehdizadeh et al., 2017; Wu et al., 2017; Jiao et al., 2018; Kaur et al., 2018; Bhatnager et al., 2019; Liu et al., 2019; Rahimi and Mohammadi, 2019; Munawar Lone et al., 2020; Ashraf et al., 2021), eight studies of Caucasian patients (Diamanti-Kandarakis et al., 1999; Marszalek et al., 2001; Cousin et al., 2004; Kahsarmiller et al., 2004; Bendlová et al., 2007; Ferk et al., 2007; Martinez-Garcia et al., 2012; Abu-Hijleh et al., 2016), and one study of mixed patients (Echiburú et al., 2008), which included 4860 PCOS cases and 4043 controls overall. All studies extracted DNA from peripheral blood, and PCR, PCR-RFLP, PCR-LDR, and TaqMan assays were used. The genotype distributions among the controls of all studies were consistent with HWE in most studies. With regard to the quality of the included studies, the Q-Genie scores were of medium quality, with an average Q-Genie score of 40.88 (range 38–44).

**TABLE 1:** The characteristics of included studies for this meta-analysis.

First Author, year	Country	Ethnicity	PCOS Diagnostic Criteria	Genotyping Method	Sample Size	Genotypes (Mtmt/Mtwt/wtwt)		*p* value for HWE	Q-genie Score
					Case/control	Case	Control		
CYP17A1 rs743572 (T/C)									
(Li et al., 2015)	China	Asian	Rotterdam	PCR-RFLP	318/306	21/139/158	28/141/137	0.148	42
(Unsal et al., 2009)	Turkey	Asian	Rotterdam	PCR-RFLP	44/50	10/19/15	6/24/20	0.361	41
(Rahimi and Mohammadi, 2019)	Iran	Asian	Rotterdam	PCR-RFLP	50/109	0/15/35	0/17/92	<0.001	40
(Park et al., 2008)	Korea	Asian	Rotterdam	TaqMan	133/99	32/61/40	33/41/25	0.213	40
(Munawar Lone et al., 2020)	Pakistan	Asian	Rotterdam	PCR-RFLP	204/100	4/112/88	2/12/86	<0.001	39
(Marszalek et al., 2001)	Poland	Caucasian	NIH/NICHD	PCR-RFLP	55/56	11/27/17	7/29/20	0.648	41
(Kaur et al., 2018)	India	Asian	Rotterdam	PCR-RFLP	250/250	25/118/107	10/94/146	0.001	39
(Kahsarmiller et al., 2004)	United States	Caucasian	NIH/NICHD	PCR-RFLP	259/161	38/142/79	17/94/50	0.105	41
(Echiburú et al., 2008)	Chile	Mixed	NIH/NICHD	PCR-RFLP	159/93	19/81/59	14/36/43	0.428	42
(Diamanti-Kandarakis et al.,1999)	Greece	Caucasian	NIH/NICHD	PCR-RFLP	50/50	8/58/34	0/56/44	0.007	39
(Dasgupta et al., 2014)	India	Asian	Rotterdam	PCR-RFLP	60/54	19/26/15	14/22/18	0.344	41
(Banerjee et al., 2016)	India	Asian	Rotterdam	PCR-RFLP	75/73	22/33/20	20/35/18	0.585	41
(Ashraf et al., 2021)	India	Asian	Rotterdam	PCR-RFLP	394/306	70/209/115	42/156/108	0.053	42
(Tan et al., 2005)	China	Asian	Rotterdam	PCR-RFLP	118/106	40/66/12	30/55/21	0.033	38
(Wu et al., 2017)	China	Asian	Rotterdam	PCR-RFLP	260/237	61/109/90	52/104/81	0.051	43
CYP19A1 rs2414096 (G/A)									
(Munawar Lone et al., 2020)	Pakistan	Asian	Rotterdam	PCR-RFLP	204/100	48/120/36	8/18/74	<0.001	39
(Kaur et al., 2018)	India	Asian	Rotterdam	PCR-RFLP	250/250	9/20/221	2/26/222	<0.001	39
(Mehdizadeh et al., 2017)	Iran	Asian	Rotterdam	PCR-RFLP	70/70	10/31/29	17/37/16	0.051	41
(Jin et al., 2009)	China	Asian	Rotterdam	PCR-RFLP	386/298	63/183/140	77/149/72	<0.001	42
(Jiao et al., 2018)	China	Asian	NIH/NICHD	PCR-LDR	350/312	77/174/99	75/131/106	0.801	44
SHBG rs6259 (G/A)									
(Abu-Hijleh et al., 2016)	Bahrain	Caucasian	Rotterdam	TaqMan	242/238	0/20/222	0/27/211	<0.001	41
(Martinez-Garcia et al., 2012)	Spain	Caucasian	NIH/NICHD	TaqMan	281/142	2/50/229	1/23/118	0.85	41
(Liu et al., 2019)	China	Asian	NIH/NICHD	PCR-RFLP	261/217	21/109/131	9/70/138	0.008	41
(Bhatnager et al., 2019)	India	Asian	Rotterdam	PCR-RFLP	200/200	1/23/176	0/22/178	0.875	43
(Bendlová et al., 2007)	Czech Republic	Caucasian	Rotterdam	PCR-RFLP	248/109	2/40/206	0/16/93	0.789	41
(Dai et al., 2015)	China	Asian	Rotterdam	TaqMan	116/148	5/22/89	17/35/96	0.003	40
(Ferk et al., 2007)	Slovenia	Caucasian	Rotterdam	TaqMan	123/110	0/17/106	2/17/91	<0.001	40
(Cousin et al., 2004)	France	Caucasian	NIH/NICHD	PCR	154/149	2/56/96	0/48/101	0.053	40

Abbreviationsmt, Mutant type; wt, Wild type; HWE, Hardy-Weinberg equilibrium; PCR-RFLP, polymerase chain reaction-restriction fragment length polymorphism.

### Quantitative Analysis

For a quantitative analysis, at least two studies are required for each polymorphism. For there were few cohort studies on specific loci polymorphisms (such as only one study on CYP17A1 loci -600, CYP17A1 loci +723, CYP17A1 loci +2612, CYP17A1 loci +4259, CYP17A1 loci +4444, CYP17A1 loci +4994, CYP19A1 rs2236722, CYP19A1 rs700518, SHBG rs13894, SHBG rs858521, SHBG rs1799941 and SHBG rs6527; two studies on CYP19A1 rs700519; three studies on SHBG rs727428), to make the conclusions more reliable, we only included loci with no less than five cohort studies for quantitative analysis.

### Association Between the CYP17A1 (rs743572) Polymorphisms and PCOS Risk

**FIGURE 2:**
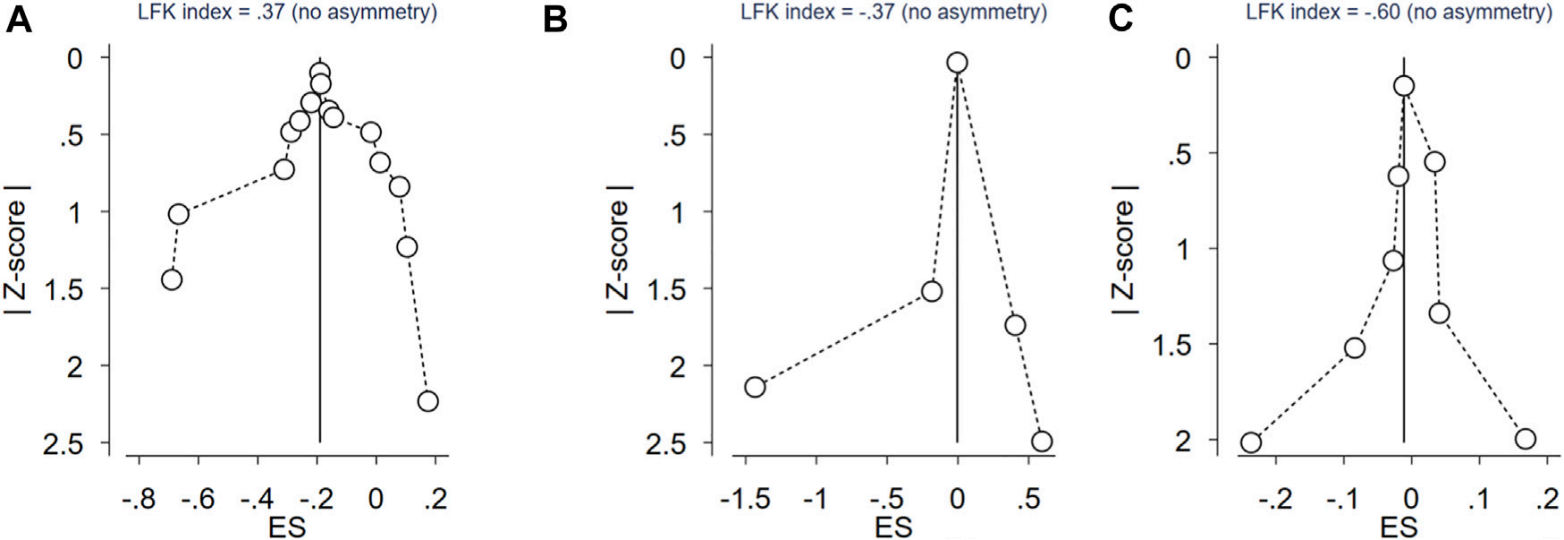
Doi plot of rs743572 of CYP17 gene polymorphisms, rs2414096 of CYP19 gene polymorphisms, rs6529 of SHBG gene polymorphisms and PCOS risk. **(A)** Doi plot of rs743572 of CYP17 gene polymorphisms and PCOS risk; **(B)** Doi plot of rs2414096 of CYP19 gene polymorphisms and PCOS risk; **(C)** Doi plot of rs6529 of SHBG gene polymorphisms and PCOS risk.

In the current meta-analysis, 15 case-control studies involving 2429 PCOS patients and 2050 control women were included to estimate the relationship between the CYP17A1 (rs743572) polymorphisms and PCOS risk. As shown in Table 2, the CYP17A1 rs743572 polymorphisms was found to be negatively associated with PCOS risk under dominant model (*p* = 0.017, OR = 0.83, 95%CI 0.72–0.97, *I* ^*2*^ = 74.80%, *P*_*heterogeneity*_ = 0.000) in the general population. In subgroup analyses by ethnicity, however, no significant association between the CYP17A1 rs743572 polymorphisms and the PCOS susceptibility was found in the Asian (*p* = 0.054, OR = 0.83, 95%CI 0.68–1.00), Caucasian (*p* = 0.252, OR = 0.87, 95%CI 0.72–1.09) and mixed (*p* = 0.148, OR = 0.80, 95%CI 0.60–1.08) population. We evaluated the overall potential publication biases with LFK index = 0.37 and Doi plots showing no asymmetry (Figure 2A).

**TABLE 2:** Meta-analysis of the association gene polymorphism and the polycystic ovary syndrome risk.

Compared genotype	No. of Studies	Sample Size	OR (95%CI)	*P*	Heterogeneity		LFK Index
		PCOS/control			*I* ^*2*^ (%)	^a^ *P*	
CYP17A1 rs743572 Dominant model (TC + CC vs. TT)							
Overall	15	2429/2050	***0.83(0.72–0.97*** *)*	***0.017***	74.80%	0.000	0.37
Asian	11	1906/1690	0.83 (0.68–1.00)	0.054	81.50%	0.000	0.37
Caucasian	3	364/267	0.89 (0.72–1.09)	0.252	0.00%	0.588	-0.30
Mixed	1	159/93	0.80 (0.60–1.08)	0.148	—	—	0.00
CYP19A1 rs2414096 Dominant model (GA + AA vs. GG)							
Overall/Asian	5	1260/1030	0.87 (0.54–1.41)	0.578	95.90%	0.000	-0.37
SHBG rs6259 Dominant model (GA + AA vs. GG)							
Overall	8	1625/1313	0.99 (0.94–1.05)	0.752	60.90%	0.012	-0.60
Asian	3	577/565	0.98 (0.81–1.18)	0.794	85.50%	0.001	-0.30
Caucasian	5	1048/748	1.01 (0.97–1.05)	0.704	0.00%	0.479	-3.86

OR, odds ratio; CI, confidence interval. All investigated polymorphisms contain a wild type allele (wt) and a mutant type allele (mt), The pooled ORs, were performed for dominant model (mtmt + wtmt vs. wtwt). The values in bold and inclined results are statistically significant between cases and controls.

a p Q-test, the *p* value for heterogeneity test.

### Association Between the CYP19A1 (rs2414096) Polymorphisms and PCOS Risk

In the current meta-analysis, five case-control studies involving 1260 PCOS patients and 1030 control women were included to estimate the relationship between the CYP19A1 (rs2414096) polymorphisms and PCOS risk. All of the five studies were conducted in the Asian population (Table 2), no significant association between the CYP19A1 rs2414096 polymorphisms and the PCOS susceptibility was found under dominant model (*p* = 0.578, OR = 0.87, 95%CI 0.54–1.41, *I* ^*2*^ = 95.90%, *P*_*heterogeneity*_ = 0.000). We evaluated the overall potential publication biases with LFK index = -0.37 and Doi plots showing no asymmetry (Figure 2B).

### Association Between the SHBG (rs6529) Polymorphisms and PCOS Risk

In the current meta-analysis, we explored the relationship between the SHBG (rs6529) polymorphisms and PCOS risk. As shown in Table 2, for SHBG rs6529 polymorphisms and the PCOS susceptibility, eight case-control studies involving 1625 PCOS patients and 1313 control women were included, with no significant association found under dominant model (*p* = 0.752, OR = 0.99, 95%CI 0.94–1.05, *I* ^*2*^ = 60.90%, *P*_*heterogeneity*_ = 0.012) in the general population. In subgroup analyses by ethnicity, no significant association between the SHBG rs6529 polymorphisms and the risk of PCOS susceptibility was found in the Asian (*p* = 0.794, OR = 0.98, 95%CI 0.81–1.18) and Caucasian (*p* = 0.704, OR = 1.01, 95%CI 0.97–1.05) population. The overall potential publication biases were evaluated with LFK index = -0.60 and Doi plots showing no asymmetry (Figure 2C).

## Discussion

Our meta-analysis included 4860 PCOS patients and 4043 control participants from 26 case-control studies to evaluate CYP17A1, CYP19A1, and SHBG gene polymorphisms. The results showed that CYP17A1 rs743572 polymorphisms were found to be negatively associated with PCOS risk under dominant model in the general population while neither CYP19A1 rs2414096 polymorphisms nor SHBG rs6529 polymorphisms was associated with PCOS susceptibility under dominant model in the general population.

The CYP17A1 gene encodes a cytochrome P450 enzyme on chromosome 10q24.3. The enzyme converts pregnenolone and progesterone into 17-hydroxyprogesterone and 17-hydroxyprogesterone, respectively, through the activity of 17-hydroxylase. These steroids are converted to dehydroepiandrosterone (DHEA) and 4-androstenedione through the activity of 17, 20-lyase (Mykhalchenko et al., 2017). An additional Sp1 transcription factor binding site is produced at position-34 (- 34T/C) of the promoter, which regulates the expression of CYP17A1 and thus the androgen levels (Sharp et al., 2004). Many studies have explored the relationship between CYP17A1 gene polymorphisms and PCOS risk, and all of them have focused on the T > C polymorphisms in the promoter region. Pusalkar et al. showed that the frequency of the C allele increases in Indian women with PCOS, which may affect their hyperandrogenic phenotype (Pusalkar et al., 2009). However, a meta-analysis published by Li et al., in 2012 showed that in the overall analyses and some subgroup analyses (by race and country), the CYP17A1 rs743572 T > C polymorphisms were not associated with PCOS risk, but a significant increase in the PCOS risk was observed in studies within HWE and in small sample studies (Li et al., 2012). Compared to the study by Li et al., our meta-analysis included more case-control studies and confirmed that the CYP17A1 rs743572 gene polymorphisms were negatively associated with the risk of PCOS under dominant model. In the current study, neither LFK index nor Doi plots showed a publication bias.

The CYP19A1 gene is located on chromosome 15q21.2 and encodes aromatase P450, which plays an important role in the synthesis of estrogens from androgens (Dadachanji et al., 2018). Aromatase activity is less in thin and obese women with PCOS, which may be further inhibited by hyperandrogenemia (Somboonporn and Davis, 2004). The hyperandrogenic follicular environment may be a key factor leading to the downregulation of the expression of aromatase in luteinized granulosa cells in women with PCOS (Yang et al., 2015). One study demonstrated hypermethylation of the promoter and decreased levels of CYP19A1 mRNA and protein in PCOS ovaries, which indicated that the expression of aromatase was inhibited (Yu et al., 2013). However, some results are contradictory. A meta-analysis by Sharma et al. showed that there was a significant association between the CYP19A1 rs2414096 gene polymorphisms and PCOS risk in non-Indian populations, while no association was found in Indian populations (Sharma et al., 2021). However, our meta-analysis found no significant association between the CYP19A1 rs2414096 gene polymorphisms and PCOS risk under dominant model. Additionally, Rahimi et al (Park et al., 2008) and Kaur et al. (Kahsarmiller et al., 2004) also found that CYP19A1 rs2236722 and rs700519 did not show significant association with PCOS. As there are few studies on CYP19A1 gene polymorphisms and all of them are on Asian people, studies at a larger scale with different ethnicities are required to confirm the relationship between CYP19 gene polymorphisms and PCOS risk in the future.

SHBG is mainly synthesized in the liver, binding androgens in a high-fidelity manner, and thereby making it biologically inaccessible to target tissues. Several polymorphisms of the SHBG gene on chromosome 17 have been shown to alter SHBG liver biosynthesis, plasma levels, and plasma clearance efficiency, thereby regulating the distribution of androgens (Hammond, 2016). In patients with PCOS, SHBG concentrations are usually low because of elevated androgen levels, and hyperandrogenemia promotes compensatory hyperinsulinemia and insulin resistance by increasing lipoprotein and reducing insulin clearance (Shorakae et al., 2018). Hyperinsulinemia and hyperandrogenemia can hinder the secretion and synthesis of SHBG in the liver (Lim et al., 2013). Women with an SHBG deficiency showed truncated SHBG synthesis and abnormal glycosylation, which led to a significant decrease in the SHBG levels and an increase in the circulating free T levels. The D327N (rs6259) SNP was the first reported SHBG gene polymorphism, and it was found to increase the half-life of the SHBG and decrease its clearance rate, resulting in an overall increase in SHBG concentration (Cousin et al., 1998). Several studies have explored the relationship between the SHBG rs6259 SNP and serum SHBG concentration and PCOS risk, but they present conflicting results (Cousin et al., 2004; Bendlová et al., 2007; Ferk et al., 2007; Martinez-Garcia et al., 2012; Dai et al., 2015; Abu-Hijleh et al., 2016; Bhatnager et al., 2019; Liu et al., 2019). Our results showed that SHBG rs6259 was not associated with PCOS risk, which is in agreement with the results of studies by Li et al. and Liao et al. (Liao and Cao, 2020; Li et al., 2021). Additionally, Liao et al. also found that a null link between the SHBG rs727428 polymorphism and the risk of PCOS was also found under any genetic models (Liao and Cao, 2020). Due to the limited sample size and a high degree of heterogeneity among study groups, the reliability and consistency of the results may be affected.

Our meta results confirmed that androgen synthesis related gene polymorphisms may affect ovarian function and thus affect the susceptibility of PCOS. Neuroendocrine dysfunction is a component of PCOS, mainly manifested by increased secretion of LH. LH excess associated with PCOS may be secondary to the peripheral events within the ovary, the mechanism of neuroendocrine dysfunction resulting in an elevated LH in PCOS may be an uncoupling of hypothalamic estradiol inhibition by elevated ovarian androstenedione, while elevated LH may cause hyperandrogenemia (Doi et al., 2005; Doi, 2008). Herein, we also tried to explore the relationship between serum LH level and androgen synthesis related gene polymorphism, but due to limited data, meta-analysis was not possible. Li (Li et al., 2015) and Wu (Wu et al., 2017) studied the relationship between serum LH and CYP17A1 gene polymorphisms, but reached a repulsive conclusion. Only Jin (Jin et al., 2009) studied the relationship between serum LH and CYP19A1 gene polymorphisms and found that in PCOS patients the serum LH of AA type was lower than that of GA or GG type.

As hyperandrogenemia is a key characteristics seen in PCOS patients, we focused on the CYP17A1 and CYP19A1 gene polymorphisms that affect androgen synthesis and the SHBG gene polymorphisms that affect SHBG synthesis to determine the relationship between gene polymorphisms and PCOS risk through a comprehensive literature search, so as to draw more comprehensive, accurate, and reliable conclusions. In addition, the studies that we included here were conducted in different countries and using different ethnic groups, making the pooled results more universal. Finally, according to the quality evaluation system, all the articles included in this study were of medium quality.

The present study has several limitations. First, in this meta-analysis, the combined OR was estimated by the number of genotypes or alleles in the case and control subjects, without adjusting for other confounding factors, which might cause bias. Second, although the study group was large, some subgroup analyses were performed with quite small sample sizes. Despite the sensitivity analysis, our results might still be affected by a type II error. Thus, future research encompassing a larger and more detailed sample set is necessary. Third, we did not search for gray literature, the publication bias might still affect the results of our meta-analysis. Fourth, some polymorphic studies have significant inter-study heterogeneity, and some studies have a deviation from the genotype distribution of the HWE. Finally, this meta-analysis does not discuss gene-environment or gene-gene interactions. However, the conclusions and limitations of this study provide some direction for the design of future studies.

## Conclusion

In summary, this meta-analysis showed that CYP17A1 rs7435721 polymorphisms might serve as a protective factor against PCOS in general populations. Given that the pathogenesis of PCOS is extremely complex, the probability that specific gene polymorphisms could significantly contribute to its development is low, and we strongly recommend further studies with a larger sample size to comprehensively explore the potential roles of gene-gene and gene-environmental interactions in the development of PCOS.

## Summary

CYP17A1 rs743572 polymorphisms were found to be negatively associated with PCOS risk under dominant model in the general population while neither CYP19A1 rs2414096 polymorphisms nor SHBG rs6529 polymorphisms was associated with PCOS susceptibility under dominant model in the general population. CYP17A1 rs7435721 polymorphisms in general populations might be protective factors against PCOS.

## Data Availability

The original contributions presented in the study are included in the article/Supplementary Material, further inquiries can be directed to the corresponding author.
